# Rare De Novo Copy Number Variants in Patients with Congenital Pulmonary Atresia

**DOI:** 10.1371/journal.pone.0096471

**Published:** 2014-05-14

**Authors:** Li Xie, Jin-Lan Chen, Wei-Zhi Zhang, Shou-Zheng Wang, Tian-Li Zhao, Can Huang, Jian Wang, Jin-Fu Yang, Yi-Feng Yang, Zhi-Ping Tan

**Affiliations:** 1 Department of Cardiothoracic Surgery, the Second Xiangya Hospital of Central South University, Changsha, Hunan Province, China; 2 The Clinical Center for Gene Diagnosis and Therapy of the State Key Laboratory of Medical Genetics, the Second Xiangya Hospital of Central South University, Changsha, Hunan Province, China; Emory University School Of Medicine, United States of America

## Abstract

**Background:**

Ongoing studies using genomic microarrays and next-generation sequencing have demonstrated that the genetic contributions to cardiovascular diseases have been significantly ignored in the past. The aim of this study was to identify rare copy number variants in individuals with congenital pulmonary atresia (PA).

**Methods and Results:**

Based on the hypothesis that rare structural variants encompassing key genes play an important role in heart development in PA patients, we performed high-resolution genome-wide microarrays for copy number variations (CNVs) in 82 PA patient-parent trios and 189 controls with an Illumina SNP array platform. CNVs were identified in 17/82 patients (20.7%), and eight of these CNVs (9.8%) are considered potentially pathogenic. Five de novo CNVs occurred at two known congenital heart disease (CHD) loci (16p13.1 and 22q11.2). Two de novo CNVs that may affect folate and vitamin B_12_ metabolism were identified for the first time. A de novo 1-Mb deletion at 17p13.2 may represent a rare genomic disorder that involves mild intellectual disability and associated facial features.

**Conclusions:**

Rare CNVs contribute to the pathogenesis of PA (9.8%), suggesting that the causes of PA are heterogeneous and pleiotropic. Together with previous data from animal models, our results might help identify a link between CHD and folate-mediated one-carbon metabolism (FOCM). With the accumulation of high-resolution SNP array data, these previously undescribed rare CNVs may help reveal critical gene(s) in CHD and may provide novel insights about CHD pathogenesis.

## Introduction

Congenital heart diseases (CHDs) are the most common birth defects and cause significant morbidity and mortality in children worldwide. The incidence of CHD has been estimated at approximately seven per 1000 newborns, which has not changed over the past several decades [1]. Pulmonary atresia (PA) is rare and is one of the most severe cardiac malformations. This condition is characterized by a completely atretic pulmonary valve and the obstruction of blood outflow from the heart to the lungs [2]. PA only accounts for 1% of all CHDs; however, despite tremendous advances in the medical care of CHD patients, PA remains a leading cause of neonatal loss, especially in infants diagnosed with PA with an intact ventricular septum (PA-IVS) [3], [4].

Rare genetic variantions have largely been ignored in the study of complex genetic disorders and other phenotypes [5]–[7], such as cardiovascular diseases, despite the fact that the genetic cause of these diseases remains largely unknown. Recent advances in microarray-based genome profiling analysis have greatly facilitated the delineation of rare de novo copy-number variations (CNVs) that underlie cardiovascular diseases. For example, rare CNVs have become a focus in the exploration of the genetic contributions to tetralogy of Fallot (TOF) [7]–[10], syndromic CHD [11], [12], thoracic aortic aneurysms and dissections [13], and long QT syndrome [14].

More than 1700 genes are thought to play integral roles in mouse cardiac development, and homologs of these genes might be candidate genes involved in CHD in humans [15]. However, only a small number of these genes have been found to carry mutations associated with CHD in humans [16]–[18]. One plausible explanation is that mutations in critical genes result in severe birth defects or are lethal to the fetus; thus, these mutations are not found in living individuals with less severe defects [19]. We hypothesized that we would more likely discover rare structural variants that encompass key genes involved in heart development in PA than in TOF or less severe types of CHD. By focusing on one of the most severe types of CHD, PA, and by genotyping 82 patient-parent trios with an Illumina SNP array platform, we detected three previously undescribed genomic lesions: 5q14.1dup (*DHFR*), 10p13dup (*CUBN*) and 17p13.2del (*CAMTA2*).

## Methods and Materials

### Patient Selection

This study was approved by the Review Board of the Second Xiangya Hospital of the Central South University. All probands and their relatives who participated in the study gave written informed consent. All participants consented to this study, and blood samples were collected after receiving written informed consent from the patient's parent or guardian on behalf of the children enrolled. After the screening of more than 6950 patients under age 14 with CHD who were admitted to the Department of Pediatric Cardiology at the Second Xiangya Hospital of Central South University, 82 PA patients and their unaffected parents were recruited to this study and began the Cardiac Biospecimen Collection procedure. The patient cohort included 30 males and 52 females from South Central China, with a mean age of 3 years old (range of 10 days to 14 years old). The whole blood samples were collected and stored in the Cardiac Genomic Bank of the Second Xiangya Hospital of the Central South University.

Twin studies with congenital malformations are one of the commonly accepted methods for the systematic study of human genetics because twins help elucidate the relationships between genetics, epigenetics, and the environment. Three twin pairs, including two pairs of monozygotic twins and one dizygotic twin pair, were included in this study (Table 1).

**Table 1:** Three Chinese pair twins with pulmonary atresia and/or tetralogy of Fallot.

Family	Case	Status	Age	Sex	CHD types
1	Twin A	dizygotic	3y	F	PA
	Twin B				PA
2	Twin A	monozygotic	6m	F	PA
	Twin B				TOF
3	Twin A	monozygotic	2y	F	PA
	Twin B				TOF

PA, pulmonary atresia; TOF, tetralogy of Fallot; y, years; m, month; F, female

Each PA patient and their unaffected parents were assessed using echocardiography, and the diagnosis of PA was further confirmed at the time of surgery. A total of 189 ethnically matched control subjects were also evaluated by echocardiography to exclude CHD.

### Cytogenetic Analysis

The peripheral blood samples from the PA patients and the parents of those patients with potentially pathogenic CNVs were collected from each individual and followed by chromosome analysis using conventional G-banding techniques at the 550 band level. A lymphocyte culture was performed for all samples according to a standard cytogenetic protocol.

### DNA Extraction

Genomic DNA was prepared from peripheral blood using a DNeasy Blood & Tissue Kit (Qiagen, Valencia, CA) on the QIAcube automated DNA extraction robot (Qiagen, Hiden, Germany).

### CNV Identification and Novelty Assessment

Genomic DNA samples from the patients and parents were adjusted to a final concentration of 50 ng/µl. The Human660W-Quad/HumanOmni1-Quad BeadChips (Illumina Inc, San Diego, USA) and Illumina BeadScan genotyping system (Beadstation Scanner) were employed to obtain the signal intensities of SNP probes. The Human660W-Quad contains nearly 660,000 SNP markers, and the HumanOmni1-Quad Beadchip contains over 1.1 million loci, including markers derived from the three HapMap studies, the 1000 Genomes Project and recently published studies [20]. GenomeStudio V2011 software was used to analyze the genotypes (human genome build 37/Hg19 was used for analysis), and the data were further analyzed with a program that included Illumina cnvPartition (V2.3.4) to call the CNVs. To assess the novelty of high-confidence CNVs, we compared their coordinates to known CNVs in the database of Genomic Variants and DECIPHER (Database of Chromosomal Imbalance and Phenotype in Humans Using Ensembl Resources). Our raw data for public release have been approved and assigned GEO accession numbers GSE56422 study at: http://www.ncbi.nlm.nih.gov/geo/query/acc.cgi?acc=GSE56422. The entries are scheduled to be released on Apr 02, 2014.

### Real-time Quantitative PCR Validation

For eight potentially pathogenic CNVs, at least two primer sets were designed within the boundaries of the CNV region (primers are listed in the Table S1). Real-time quantitative PCR (qPCR) was performed using a 7500 Fast Real-Time PCR system (Applied Biosystems, Foster City, California) to validate variable copy numbers. PCR reactions were prepared using the SYBR Premix Ex Taq II PCR reagent kit (TaKaRa Bio, Dalian, China) according to the manufacturer's instructions. Primer pairs were designed using the online PrimerQuest tool from Integrated DNA Technology (http://www.idtdna.com/Primerquest/Home/Index). Amplification levels were calculated using the 2^−ΔΔCT^ method.

## Results

Eighty-two patients with PA, including three pairs of twins, were included in this study. Among the patients, 77 PA patients with a ventricular septal defect (PA-VSD) and five PA patients with an intact ventricular septum (PA-IVS), were genotyped using SNP-based microarrays. To explore CNVs that are potentially responsible for PA and avoid overestimating the causality of CNVs, we focused on deletions/duplications (copy number 1 or 3 at the autosomes and 0 or 1 at the X chromosome in males and females, respectively) with high confidence. We identified 412 CNVs (256 copy losses and 156 copy gains) with an average of five CNVs (range of 0–28) per patient and an average length of 400,250 bp (range of 59,926–5,217,518 bp).

To identify pathogenic CNVs that encompass possible causative genes for CHD, the genes located within the CNVs were analyzed. We identified genomic lesions in 17/82 patients (20.7%), and eight of these CNVs (9.8%) are considered potentially pathogenic. The eight pathogenic or potentially pathogenic CNVs were further validated by Real-time quantitative PCR (Primers are listed in Table S1). Five de novo CNVs occurred at two known congenital heart disease (CHD) loci (16p13.1 and 22q11.2). Three rare CNVs are previously undescribed genomic aberrations: duplication of 5q14.1 (*DHFR*), duplication of 10p13 (*CUBN*) and deletion of 17p13.2 (*CAMTA2*). The results of these potentially pathogenic CNVs are summarized (Table 2).

**Table 2:** Rare CNVs identified in 82 patients with pulmonary atresia.

Patients	Loci	Copy	CNV coordinates	Size(kb)	Inheritance	Critical Genes	Status
869677	2q37.1	Loss	chr2:234255714-234415570	160	De novo	*DGKD,USP40*	UK
Family1 TwinA	3p26.3-p26.1	Loss	chr3:204652-4944387	4740	Inherited	10 genes	UK
	10p15.3-p15.1	Gain	chr10:59083-5276600	5180	Inherited	12 genes	UK
Family1TwinB	3p26.3-p26.1	Loss	chr3:38411-4944387	4906	Inherited	10 genes	UK
	10p15.3-p15.1	Gain	chr10:103934-5297659	5172	Inherited	12 genes	UK
817646	5q13.2	Loss	chr5:68930217-70573127	1643	De novo	*SMN1/SMN2*	UK
881678	5q14.1	Gain	chr5:75132315-79958945	4827	De novo	***DHFR,DMGDH*** *,PDE8B,AP3B,ARSB*	PP
894020	6p21.33	Loss	chr6:31467185-31569111	102	De novo	*NFKBIL1,LTA,TNF,NCR3*	UK
706346	6p21.33	Loss	chr6:31467627-31570907	103	De novo	*NFKBIL1,LTA,TNF,NCR3*	UK
875001	9q33.3	Gain	Chr9:127104486-127759670	655	De novo	*NR5A1,NR6A1*	UK
765647	10p13	Gain	chr10:16883466-17058324	175	De novo	***CUBN***	PP
807411	13q33.1	Gain	chr13:102902377-103345247	443	De novo	*FGF14*	UK
894147	15q26.3	Gain	chr15:99004156-99782996	779	De novo	*IGF1R,SYNM*	UK
882624	16p13.1	Gain	chr16:15395312-16270740	875	De novo	***MYH11*** *,ABCC6,NDE1*	P
791019	16p13.3	Loss	chr16:1866643-2604949	738	De novo	*ABCA3,TSC2,PKD1,TBC1D24*	UK
827979	17p13.2	Loss	chr17:4041358-5091377	1050	De novo	***CAMTA2*** *,CHRNE,GP1BA,ENO3*	PP
Family2TwinA	22q11.2	Loss	chr22:17255869-18692668	1437	De novo	*TBX1*	P
795265	22q11.2	Loss	chr22:17089407-19795050	2706	De novo	*TBX1*	P
877494	22q11.2	Loss	chr22:17101361-19801287	2700	De novo	*TBX1*	P
742265	22q11.2	Loss	chr22:17255869-19795050	2539	De novo	*TBX1*	P

P, pathogenic; UK, unknown; PP, potentially pathogenic.

### CNVs Linked to Folate and Vitamin B_12_ Metabolism

**Figure 1:**
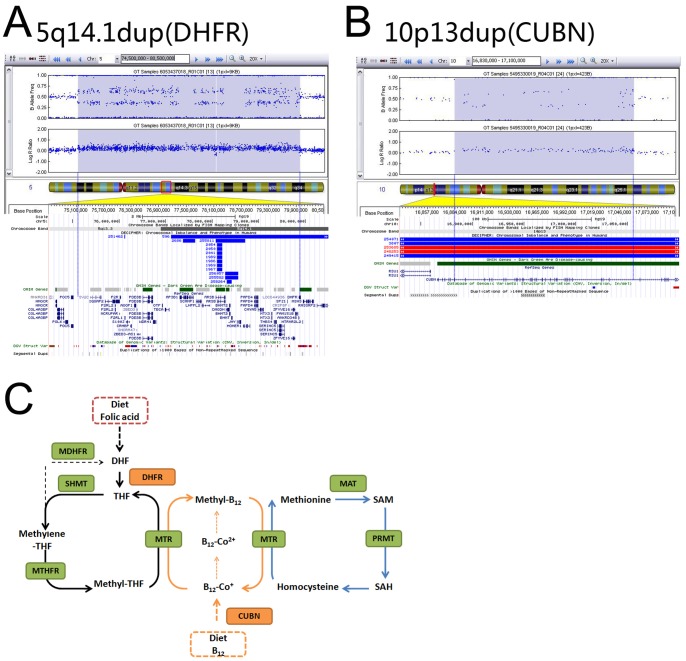
Rare CNVs related to folate and Vitamin B_12_ metabolism. (A) SNP-array shows a 4.8 Mb duplication at 5q14.1 (chr5:75132315-79958945); (B) A 175-kb duplication at 10p13 (chr10:16883466-17058324) (UCSC Genome Browser on Human GRCh37/hg19 Assembly). Log R ratio and B alle frequency are showed in the upper panel and annotated genes are listed in the lower panel. (C) Summary of Folate metabolic pathway (Methionine-Homocysteine-Folate-B12 Cycle). DHF, dihydrofolate; THF, tetrahydrofolate; DHFR, dihydrofolate reductase; MTHFR, methylenetetrahydrofolate reductase; SHMT, serine hydroxyl-methyltransferase; MAT, methionine adenosyltransferase; SAH, S-adenosylhomocysteine; SAM, S-adenosylmethionine; MTR, methionine synthase; PRMT, protein arginine methyltransferase; CUBN, Cubilin. Adapted from Lee *et al* [41].

We examined whether PA-associated CNVs encompass gene(s) that related to specific genetic pathways and identified two CNVs that encompass genes associated with folate and vitamin B12 metabolism. A 5q14.1 duplication (chr5:75132315-79958945) spanned approximately 4.8 Mb and encompassed *DHFR* (MIM 126060) and other critical genes, such as *PDE8B* (MIM 603390), *AP3B1* (MIM 603401), *ARSB* (MIM 611542), and *DMGDH* (MIM 605849). The other CNV was a 10p13 duplication that is 175 kb in size and affected only one gene, *CUBN* (MIM 602997) (Figure 1).

### CNVs in Twins with PA

**Figure 2:**
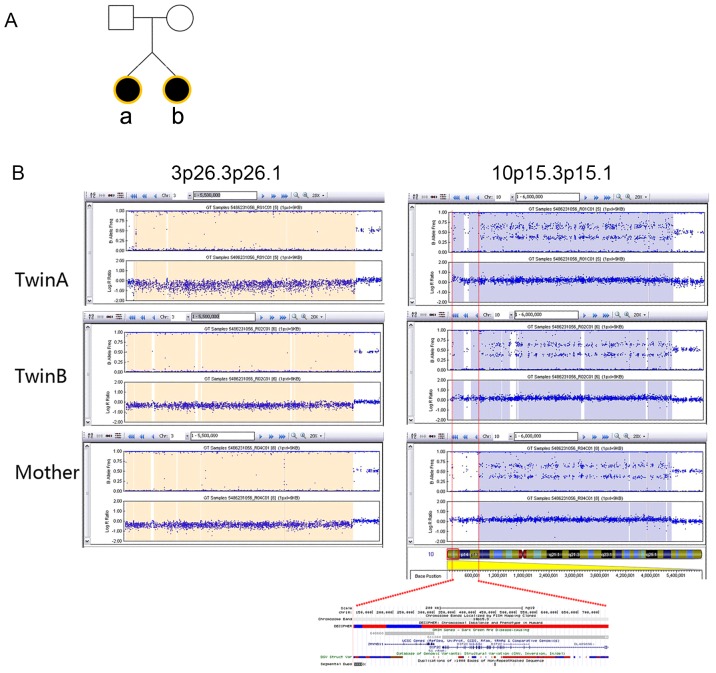
CNVs in the dizygotic twin sisters of Family 1. (A) Pedigree of family1 with dizygotic twins with PA. (B) Comparison of CNVs at 3p26.3-p26.1 and 10p15.3-p15.1 locus in twinA, twinB and the healthy mother. A minimal 620-kb duplication at 10p15.3 (chr10:103934-724229) was found in the twin sisters but not presented in the healthy mother. Annotated genes within this region are listed in the lower panel.

The patient cohort also included three pairs of twins: one pair of dizygotic twin sisters who both had PA and two monozygotic pairs who were diagnosed with PA or TOF. Identical 3p26.3-26.1 deletions and 10p15.3-p15.1 duplications were identified in the dizygotic twin sisters with PA (family 1, Figure 2). The karyotypes of the twins and their parents were normal according to traditional G-banding analysis (Figure S1). A 22q11.2 deletion (1.5 Mb) was detected in the pair of monozygotic twins from family 2 with discordant CHD subtypes, PA and TOF (Table 1).

### Other Rare de novo CNVs in PA

**Figure 3:**
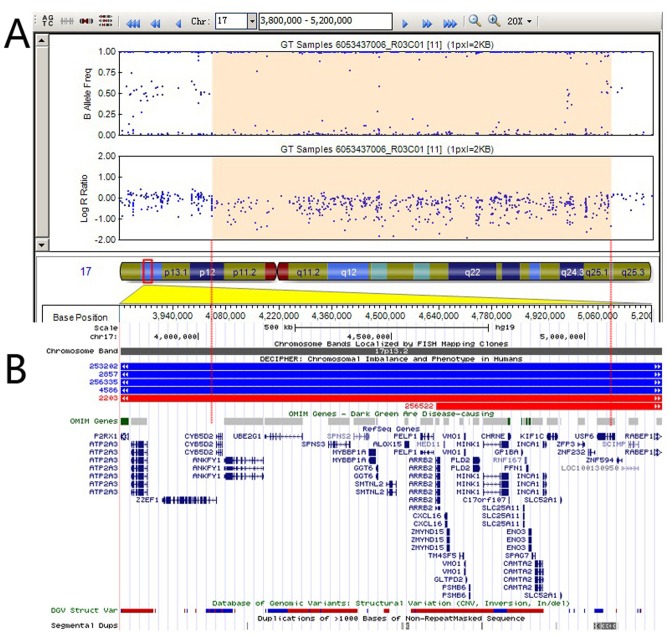
Illumina SNP-array result of the 17p13.2 region in patient 827979. (A) SNP based array shows a 1.05 Mb deletion at 17p13.2 (chr17:4041358-5091377) (Human GRCh37/hg19 Assembly). B allele frequency and Log R ratio are showed in the upper panel; (B) The lower panel shows genes mapped to the deleted region. OMIM genes are highlighted in green, DGV structure variants and Segmental Duplications are also enclosed in the lower panel.

Rare potentially pathogenic CNVs at 17p13.2del (chr17:4041358-5091377) were observed. The 1-Mb deletion at 17p13.2 spanned four OMIM morbid genes: *CHRNE* (MIM 100725), *GP1BA* (MIM 606672), *PFN1* (MIM 176610), and *ENO*3 (MIM 131370) (Figure 3). The single occurrences of five rare CNVs that were observed (three deletions at 2q37.1, 5q13.2, and 16p13.3; and two duplications at 13q33.1 and 15q26.3) might represent benign variants (Table 2). The clinical importance of two recurrent deletions at 6p21.33 remains unknown (Figure S2).

## Discussion

We have presented one of the largest genetic studies investigating the role of rare CNVs in the severe heart defect, PA. The data from 82 PA patient-parent trios and 189 controls were analyzed with a well-defined platform (Human660W-Quad/Omni1-Quad SNP-arrays). Copy number variations were identified in 17/82 patients (20.7%), and eight of these CNVs (9.8%) are considered pathogenic or potentially pathogenic. The overall CNV frequency in our study is consistent with that found in previous studies of isolated or syndromic CHD patients based on different microarray platforms using array comparative genome hybridization or SNP arrays [7], [8], [21].

Previous research suggests that the risk of CHD may be related to genetic variants in folate pathway-related genes [22]–[26]. However, genetic evidence is lacking [27]–[29]. Our study presents evidence for an association between folate-related genes and a specific subtype of CHD (PA) in humans. We have identified two potentially pathogenic CNVs linked to folate-mediated one-carbon metabolism (FOCM). One CNV is a 4.8-Mb duplication on 5q14.1 that contains *DHFR*, and another CNV is a 10p13 duplication that affects only one gene, *CUBN*. Dihydrofolate reductase (DHFR) is a key folate-metabolizing enzyme that catalyzes the reduction of dihydrofolate (DHF) to tetrahydrofolate (THF), as well as folic acid to DHF [30]. Several groups have shown that inhibiting the expression of DHFR results in embryonic lethality in zebrafish. Interestingly, the anatomic malformations observed with PA in humans are consistent with the defects in the outflow tract in the zebrafish model [31], [32]. Cubilin, encoded by *CUBN*, is a receptor expressed in the apical pole of absorptive epithelia [33], and it plays an important role in the absorption of vitamin B_12_ [34]. The interactions between folate, vitamin B_12_, and homocysteine are summarized in Figure 1C. Both potentially pathogenic CNVs are duplications, implying that the association of CHD with these genes might be dosage-sensitive. However, the underlying molecular mechanisms linking FOCM to birth defects, such as CHD, remain unknown.

Two dizygotic twin sisters of family 1 shared an identical 3p26.3-26.1 deletion and a 10p15.3-p15.1 duplication, while the karyotypes of the twins and their parents were normal at 550 band resolution (Figure S1). The 3p26.3-p26.1 deletion spanned approximately 4.9 Mb and affected more than 10 genes, including *SUMF1*, *ITPR1,* and *CRBN*. The 5.1-Mb duplication at 10p15.3-p15.1 contained several disease-causing genes, such as *KLF6*, *AKR1C2*, and *AKR1C4*. Fine mapping of these two genomic regions (3p26.3-26.1 and 10p15.3-p15.1) revealed that the twins with PA carry a larger duplication at the terminus of chromosome 10p than their healthy mother (Figure 2B). Recently, Tremblay *et al.* mapped a gene associated with ventricular aneurysms and septal defects to chromosomal loci (10p15.3-p15.2) using classic familial linkage analysis [35]. This evidence collectively suggests that the 620-kb region at chromosomal 10p15.3 (chr10:103934-724229) may represent a plausible CNV with causative genes for CHD.

Two other de novo CNVs, one with an 875-kb duplication at 16p13.1 (chr16:15395312-16270740) and the other with a 1-Mb deletion at the 17p13.2 locus (chr17:4041358-5091377), were found in only one individual with PA. Of the 12 genes contained in the 16p13.1 region, *MYH11* has been previously associated with heart disease. Mutations in *MYH11* cause thoracic aortic aneurysm/dissection, and recurrent 16p13.1 duplications confer a risk for aortic dissection [7], [13], [36]. The 1-Mb deletion at 17p13.2 covers a gene-rich region (with 30 annotated genes), suggesting that this CNV is potentially pathogenic. In this 17p13.2 deleted region, the gene *CAMTA2*, designated as calmodulin binding transcription activator 2, is part of a family of activators of the ANF promoter. Camta2 has been shown to interact with Nkx2-5 and promote cardiac hypertrophy in mice [37]. For this reason, *CAMTA2* is an appropriate candidate gene for CHD that is caused by a 17p13.2 deletion disorder. The 17p13.2 deletion is rarely reported in the literature, and thus far, only two 17p13 deletions [38], [39] and one 17p13 duplication [40] have been reported in patients with intellectual disability. A re-evaluation of this individual with the 17p13.2 deletion identified mild intellectual disability, hypotonia, and subtle facial features, raising the possibility of a previously unrecognized microdeletion disorder.

In summary, we identified eight rare de novo CNVs (9.8%) in patients with PA including three previously undescribed CNVs: 5q14.1dup (*DHFR*), 10p13dup (*CUBN*) and 17p13.2del (*CAMTA2*). Our results suggest that rare CNVs likely contribute significantly to the genetic basis of cardiovascular diseases, such as PA. Our study provides evidence for the association of CHD with FOCM. The discovery of previously undescribed rare CNVs in patients with CHD helps elucidate critical gene(s) for CHD and may provide new insights into understanding the pathogenesis of CHD.

## Supporting Information

Figure S1 **G-banded karyotype analysis of the twin family 1.** Both individuals (Twin A and Twin B) have normal karyotypes.
(TIF)

Figure S2 **Illumina SNP-array result of the 6p21.33 deletions in patient 894020 and patient 706346.**
(TIF)

Table S1 **Primers for Real-time Quantitative PCR Validation.**
(DOCX)
