# Supplementary figures and images for: Rare De Novo Copy Number Variants in Patients with Congenital Pulmonary Atresia
Source: PLoS One. 2014 May 14;9(5):e96471. doi: 10.1371/journal.pone.0096471 (PMC4020819; doi:10.1371/journal.pone.0096471)

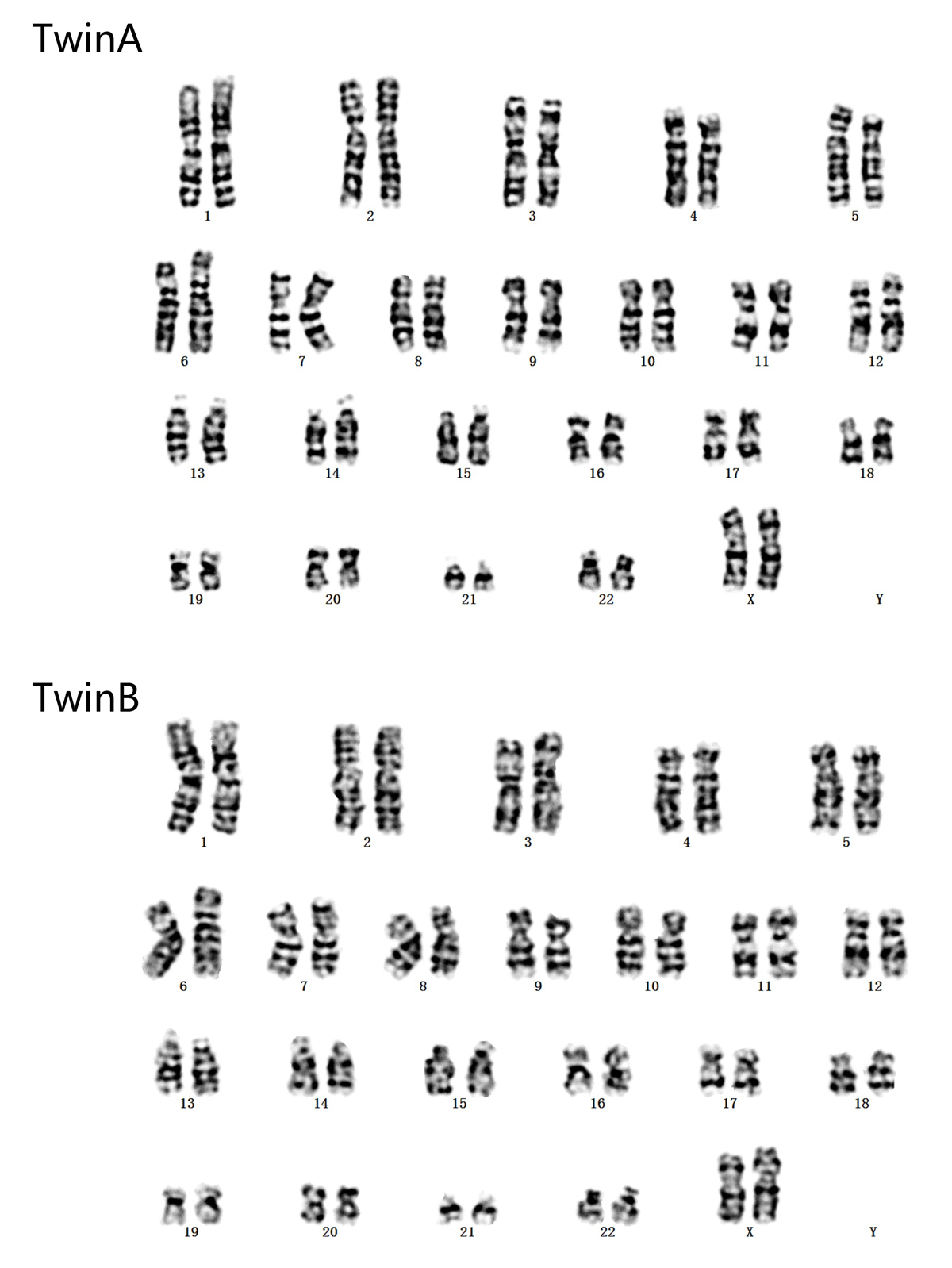

Supplement: Figure S1 — G-banded karyotype analysis of the twin family 1. Both individuals (Twin A and Twin B) have normal karyotypes. (TIF) [file pone.0096471.s001.tif]

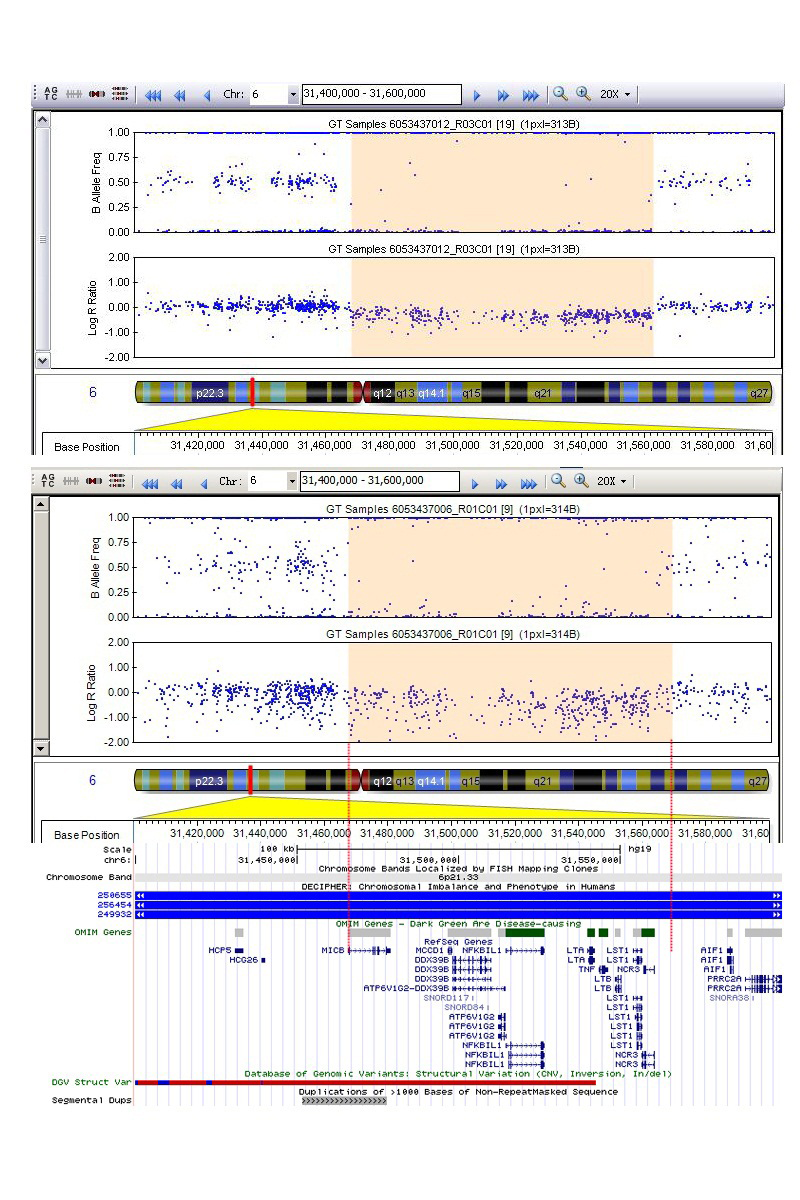

Supplement: Figure S2 — Illumina SNP-array result of the 6p21.33 deletions in patient 894020 and patient 706346. (TIF) [file pone.0096471.s002.tif]
