# Supplementary material for: Rare De Novo Copy Number Variants in Patients with Congenital Pulmonary Atresia
Source: PLoS One. 2014 May 14;9(5):e96471. doi: 10.1371/journal.pone.0096471 (PMC4020819; doi:10.1371/journal.pone.0096471)
Supplement: Table S1 — Primers for Real-time Quantitative PCR Validation. (DOCX) [file pone.0096471.s003.docx]

| CNVs | Gene | Primer sequence for qPCR(5’-3’) |
| --- | --- | --- |
| **Pathogenic or potentially pathogenic** |  |  |
| 5q14.1 duplication | DHFRF | CAGATTATTTGACTAGTATGGATGG |
|  | DHFRR | CTTTCCAGAAGTCTAGATGATGC |
|  | DMGDHF | TGACTTATTTATATGCTCTCACTGG |
|  | DMGDHR | TATCAGACTTGCTACCACCC |
| 10p13 duplication | CUBNF1 | TGATATTAAGAGACAGGCACTGG |
|  | CUBNR1 | GTCGTCATATTTGGATGCACAC |
|  | CUBNF2 | GGCCTGTTCTCTTCTAAGACTAAAT |
|  | CUBNR2 | GCACAGCCGAAGCATTTC |
| 16p13.1 duplication | MYH11F | TTCTACAAGCAAACCCGATTCT |
|  | MYH11R | GTAAGTAGCAAAGCCACATGGA |
|  | ABCC6F | CCTCATTCATCCTACTGTGTGG |
|  | ABCC6R | TCCCAGCTGCTGCTTTG |
| 17p13.2 deletion | CAMTA2F | CCCAGGGTAGCACACTAAAC |
|  | CAMTA2R | AGCGAGGAAGACACTCCA |
|  | ENO3F | GCAGAAGCTCTCATCCTTTCT |
|  | ENO3R | CTGCTCCCGCCTTACAC |
| 22q11.2 deletion | TBX1F | GGGAAACTTCTCAAAGGCACT |
|  | TBX1R | AGGAGGAGCTGTGGAAGG |
|  | BCL2L13F | CCACCACACCCAGCTAATTT |
|  | BCL2L13R | CTGAACACTGGAGAAGTGTGAC |
| **Unknown clinical importance** |  |  |
| 3p26.3-p26.1 deletion | CNTN4F | AGTGGTACTCTCCCTTGATATCTT |
|  | CNTN4R | AGACACAGTGCTTCTTGTTCTT |
|  | TRNT1F | TTTCAGTCGGCTGGGATT |
|  | TRNT1R | TTTGACTCACCCTGGCA |
| 10p15.3-p15.1 duplication | GTPBP4F | GGAGGGAGAGGGAAAGAAATTG |
|  | GTPBP4R | TCAAATCAGCATAGAACGGATGA |
|  | KLF6F | TTCCAGGAGCTCCAGATCG |
|  | KLF6R | CGGCTGCGTTTACCTGTT |
| 6p21.33 deletion | LTAF | GCATCTTGCCCACAGCA |
|  | LTAR | GGTAGGAGGAGAGCTGGTG |
|  | TNFF | AGGACAGGAACCGGATGT |
|  | TNFR | CTCAGCTTGAGGGTTTGCT |
